# The effect of acupuncture on pain and swelling of arthritis animal models: A systematic review and meta-analysis

**DOI:** 10.3389/fgene.2023.1153980

**Published:** 2023-04-11

**Authors:** Wei-Lien Yu, Seung-Nam Kim

**Affiliations:** College of Korean Medicine, Dongguk University, Goyang, Republic of Korea

**Keywords:** acupuncture, electroacupuncture, arthritis, animal models, systematic review, meta-analysis

## Abstract

Arthritis, the inflammation of joints, attributes to the patient’s pain, joint deformation, and limited range of motion. Emerging studies have shown the effects of acupuncture on different types of arthritis. We aimed to assess the effects of acupuncture on arthritis animal models and summarize the related mechanisms. We retrieved studies that met our criteria from PubMed, MEDLINE, EMBASE and the Research Information Service System. The quality assessment was evaluated by using the Systematic Review Centre for Laboratory Animal Experimentation’s risk of bias tool. The pain withdrawal latency, pain withdrawal threshold, and paw volume data were digitized using Engauge Digitizer software. The meta-analysis was performed, and the figures were generated using RevMan software. The meta-analysis of data from 21 animal studies revealed that acupuncture increased tolerance to pain stimuli, and reduced swelling in arthritis animals. Although the number of included studies is insufficient, the results suggest acupuncture to be effective in improving arthritis-induced inflammation and pain by regulating the nervous and immune system.

## 1 Introduction

Arthritis is defined as the inflammation of one or more joints. It can cause cartilage and bone damage, pain, and physical disability. The most common types of arthritis are rheumatoid arthritis (RA), osteoarthritis (OA) and gouty arthritis (GA). RA is one of the most common chronic inflammatory diseases. The current criteria require one or more long-lasting swollen joints in the absence of other causes (Smolen et al., 2016). The joint swelling of RA is characterized by synovial membrane inflammation. Human RA is heterogeneous; the presence of serological markers like autoantibodies against IgG or against citrullinated peptides are associated with more severe subtypes of RA. The disease-modifying antirheumatic drugs are the first-line treatment for RA, aiming at reducing destructive inflammation; non-steroidal anti-inflammatory drugs (NSAIDs) are prescribed to reduce patients’ pain and stiffness. OA is a degenerative arthritis, defined by the anatomical and/or physiological abnormalities in the joint (Kuyinu et al., 2016). Pain medication and lifestyle modification is recommended for conservative treatment. Surgical intervention is considered for severe OA. Gouty arthritis is a metabolic arthropathy. The deposition of monosodium urate (MSU) crystals in joints and surrounding tissues leads to acute attacks and persistent low-grade inflammation (Wilson and Saseen, 2016). Termination of pain and prevention of recurrent attacks are the goals of GA treatment. NSAIDs, corticosteroids and colchicine, are the main anti-inflammatory medication used to treat acute gout.

Acupuncture is a widely used traditional medicine practice (Hao and Mittelman, 2014), and arthritis is one of the most frequent diseases which patients visit acupuncture clinic for. A Cochrane review on acupuncture for peripheral osteoarthritis summarized acupuncture to be effective than sham acupuncture group (Manheimer et al., 2010); while the effectiveness of acupuncture in rheumatoid arthritis and gouty arthritis is uncertain (Casimiro et al., 2005; Lu et al., 2016). There are already systemic reviews on the efficacy of acupuncture treatment in humans (Lee et al., 2020; Li et al., 2022). Yet, no related systematic reviews on animal models have been published. Thus, the efficacy of acupuncture on animal arthritis models is inconclusive, and the working mechanisms remains controversial, as well. This systemic review aims to evaluate the patterns and consistencies of acupuncture treatment in rat arthritis models and identify areas where further research is needed.

The pain sensation of animals is inferred from pain-like behaviors like the withdrawal of hindlimbs from a stimulus (Deuis et al., 2017). The pain withdrawal latency (PWL) and pain withdrawal threshold (PWT) data were collected from the included studies to evaluate acupuncture-induced analgesia in arthritis animals. The percentage increase in paw volume was used as an indicator of inflammation. In this study, we conducted a systematic review and meta-analysis using the data described above. The study characteristics and the suggested mechanisms demonstrated in the included studies were summarized.

## 2 Materials and methods

### 2.1 Search strategy

We included the English studies investigating the effect of acupuncture on arthritis animal models. EMBASE, MEDLINE, PubMed, and Research Information Service System were searched from inception until August 2022 using the following terms: “mouse (mice)” or “rat (rats)” or “animal (animals)”, “acupuncture (electroacupuncture),” and “arthritis”.

### 2.2 Inclusion/exclusion criteria

Studies were included based on the following criteria: subjects (animal models of arthritis), interventions (acupuncture, limited to manual acupuncture and electroacupuncture, as the main intervention), and outcomes (PWL, PWT and percentage increase in paw volume). PWL and PWT were used as the main outcomes to evaluate the analgesic effect of acupuncture. The percentage increase in paw volume was used to indicate the degree of inflammation. Those without access to the full text articles, or those written in languages other than English were excluded in the present study.

### 2.3 Data extraction

Two authors (Yu and Kim) independently extracted the data. The first author, publication year, animal species, type of arthritis, arthritis model, type of acupuncture and the corresponding parameters, the target outcomes (PWT, PWL, paw volume or percentage increase in paw volume) were retrieved to evaluate the effect size of acupuncture on arthritis. The mean values and standard deviation were measured using the Engauge Digitizer software version 12.1.

### 2.4 Quality assessment

The risk of bias was assessed using Systematic Review Centre for Laboratory Animal Experimentation’s risk of bias (SYRCLE’s RoB) tool (Hooijmans et al., 2014). The SYRCLE’s RoB tool contains 10 entries related to selection bias (allocation concealment, baseline characteristics and sequence generation), performance bias (blinding and random housing), detection bias (blinding and random outcome assessment), attrition bias (incomplete outcome data), reporting bias (selective outcome reporting) and other bias (other sources of bias). Each entry was marked as “Low risk of bias,” “High risk of bias” or “Unclear.” The two authors (Yu and Kim) independently evaluated the RoB score of the included studies. Review Manager (RevMan) version 5.4 software (The Cochrane Collaboration, 2020) was used to generate the risk of bias figure.

### 2.5 Statistics

PWL and PWT were used as nociceptive threshold indicators (Deuis et al., 2017). PWL is defined as the time taken by the experimental animals to withdraw their paws from the heat stimuli (thermal hyperalgesia); PWT is defined as the minimal weight to elicit a withdrawal reflex (mechanical allodynia). The mean percentage increase in paw volume was calculated by “[mean paw volumes of disease (or acupuncture groups)/mean paw volume before disease induction - 1] *100”. PWL, PWT and percentage increase in paw volume were considered as continuous data. The standardized mean differences (SMDs) were estimated based on a fixed-effect model. The forest plot and funnel plots were made using RevMan version 5.4 software (The Cochrane Collaboration, 2020). The 95% confidence interval (CI) was used, and *p*-value <0.05 was considered statistically significant. The study heterogeneity was assessed using chi-squared and *I* ^*2*^ statistics.

## 3 Results

### 3.1 Study inclusion

**FIGURE 1:**
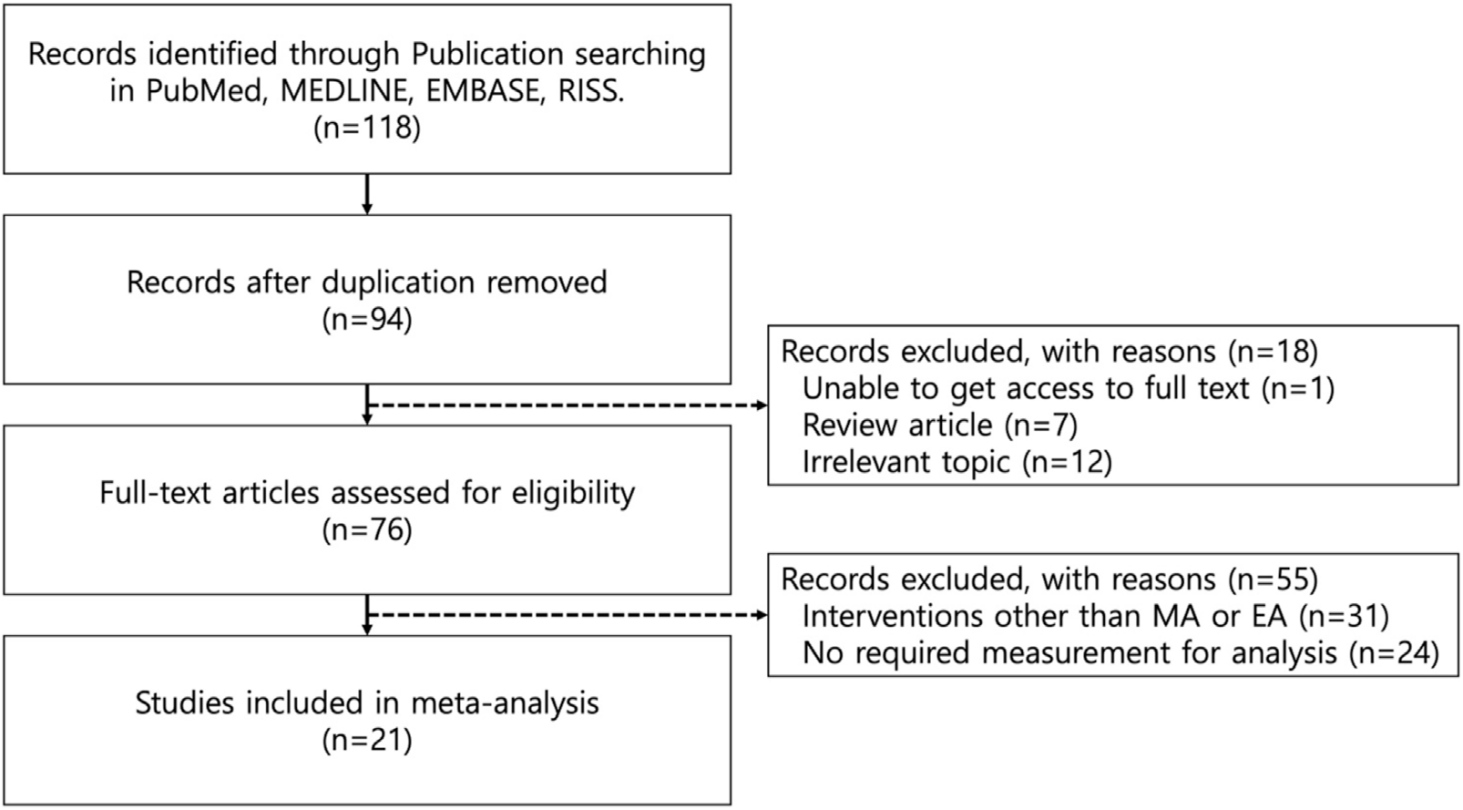
Flow diagram for Inclusion and Exclusion Process.

Among the 118 initially identified studies written in English, 94 studies left after removing duplicates. Through title and abstract screening, 7 review articles, 12 research papers of irrelevant topics and 1 study that did not provide a full text article were excluded. Full-text screening ruled out 31 studies where interventions other than MA or EA were used and 24 studies that did not include PWL, PWT, paw volume or percentage increase in paw volume measurements. A final total of 21 studies were included in the present study. The study flow is visualized in Figure 1.

### 3.2 Quality assessment

**FIGURE 2:**
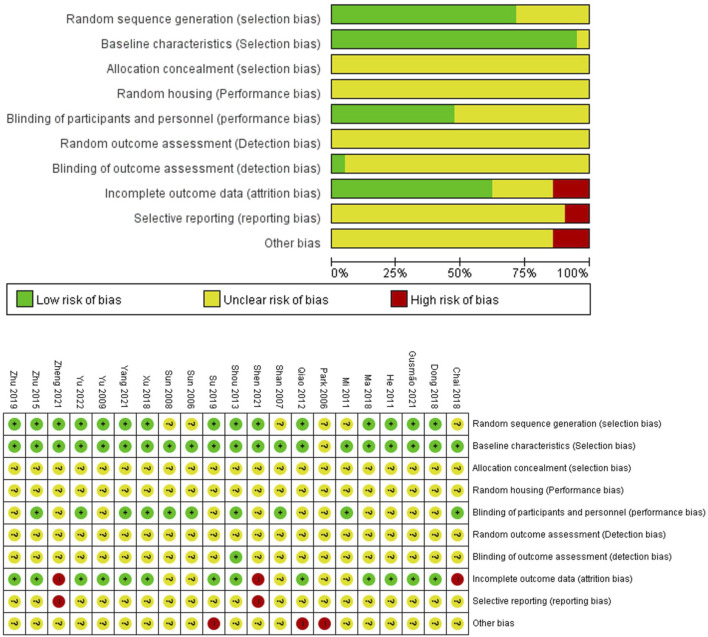
Quality assessment.

The quality assessments of the 21 studies are summarized in Figure 2. 13 studies were rated as having a low risk for the sequence generation bias, as they mentioned the rats were randomly grouped (Yu et al., 2009; He et al., 2011; Qiao et al., 2012; Shou et al., 2013; Zhu et al., 2015; Dong et al., 2018; Ma et al., 2018; Xu et al., 2018; Su et al., 2019; Zhu et al., 2019; Gusmao et al., 2021; Shen et al., 2021; Yang et al., 2021; Zheng et al., 2021; Yu et al., 2022); the remaining 8 studies did not mention the criteria for grouping. All the studies started with rats of similar weights, and they were kept under similar environmental conditions. The baselines of groups were similar except in 1 study (Park et al., 2006); the initial PWL, PWT or the paw volume values after disease induction were similar. 10 studies informed that the behavioral tests were taken by examiners blinded from the experiments (Sun et al., 2006; Shan et al., 2007; Sun et al., 2008; Mi et al., 2011; Shou et al., 2013; Zhu et al., 2015; Chai et al., 2018; Xu et al., 2018; Yang et al., 2021; Yu et al., 2022). The statistical analyses were taken in blind manner in one study (Shou et al., 2013). 4 studies were evaluated as having a high risk of bias in the “incomplete outcome data” domain: the total number of rats used for the experiments was not given, while the number of rats for each experiment differed without any explanation for such variation (Sun et al., 2008; Chai et al., 2018; Shen et al., 2021; Zheng et al., 2021). All studies included experimental results for their statements, yet we were unable to judge whether these experiments were sufficient for the conclusion. 3 studies had a high risk of other bias: one study did not provide sufficient information for EA (Su et al., 2019); two studies did not mention how deep they inserted at each acupoint (Park et al., 2006; Qiao et al., 2012). None of the articles provided information regarding the allocation concealment, random housing, and random out-come assessment domains.

### 3.3 Study characteristics

The main characteristics of each included study are summarized in Table 1. All the 21 studies used rats; among them, 6 used Wistar and the rest used Sprague–Dawley. For the reagents used for disease induction, 17 studies used complete Freund’s adjuvant (CFA), 2 used incomplete Freund’s adjuvant (CIA) for RA induction; other reagents like MSU and monosodium iodoacetate (MIA) were used for GA and OA induction, respectively. Different sites were used for the reagent injection, while ankle joint (n = 9) and foot pad (*n* = 6) were the most injected sites. EA were preferred over MA; most studies set the parameter to 2 Hz (*n* = 6) or alternate 2/100 Hz (*n* = 7) with the current ranged from 1 mA to 3 mA. Acupoints located below the knee joint were mostly used (GB34, GB39, SP6, ST35, ST36, ST44) (Lim, 2010); the remaining acupoints are located at the back (BL23, BL60, EX-B2), hip joint (GB30), and forelimb (LI4, LI11). Among these acupoint, ST36 gave the highest count (*n* = 16).

**TABLE 1:** Study characteristics.

Author	Year	Animal species	Disease	Reagent	Site of injection	Intervention	Acupoint	Parameter
Park et al	2006	Male Sprague–Dawley	RA	CFA	foot pad	EA	ST36 SP6	2 Hz, 1-2-3 mA
Sun et al	2006	MaleSprague–Dawley	RA	CFA	ankle joint	EA	GB30GB34	100/2 Hz,1-2-3 mA
Shan et al	2007	MaleSprague–Dawley	RA	CFA	ankle joint	EA	GB30GB34	2/100 Hz,1-2-3 mA
Sun et al	2008	MaleSprague–Dawley	RA	CFA	ankle joint	EA	GB30GB34	2/100 Hz,1-2-3 mA
Yu et al	2009	MaleSprague–Dawley	RA	CFA	ankle joint	EA	ST36	2/100 Hz,0.5–1.0-1.5 mA
He et al	2011	MaleWistar	RA	CFA	foot pad	EA	ST36GB39 BL23	2 Hz,6–7 mA
Mi et al	2011	MaleSprague–Dawley	RA	CFA	ankle joint	EA	GB30GB34	4/60 Hz,1 mA
Qiao et al	2012	MaleWistar	RA	CFA	toe	EA	EX-B2	2/100 Hz,1 mA
Shou et al	2013	MaleSprague–Dawley	RA	CFA	ankle joint	EA	ST36BL60	2/100 Hz,1-2-3 mA
Zhu et al	2015	MaleSprague–Dawley	RA	CIA	tail base	EA	ST36GB39	2 Hz,6–7 mA
Dong et al	2018	MaleSprague–Dawley	RA	CFA	foot pad	EA	ST36BL60	2 Hz,2 mA
Xu et al	2018	MaleWistar	RA	CFA	foot pad	MA	ST36	twisting
Su et al	2019	MaleSprague–Dawley	RA	CFA	hind paw	EA	ST36GB39	2 Hz
Zhu et al	2019	MaleSprague–Dawley	RA	CFA	foot pad	EA	ST36GB39	2 Hz,6–7 mA
Gusmão et al	2021	MaleWistar	RA	CIA	tail base	EA	LI4 LI11ST36 ST44	10 Hz3 mA
Yang et al	2021	MaleWistar	RA	CFA	hind paw	MA	ST36	twisting
Zheng et al	2021	MaleSprague–Dawley	RA	CFA	ankle joint	MA	ST36	lifting-thrusting,and twisting
Yu et al	2022	MaleWistar	RA	CFA	foot pad	MA	ST36	Twisting
Chai et al	2018	MaleSprague–Dawley	GA	MSU	ankle joint	EA	ST36BL60	2/100Hz,1–2 mA
Ma et al	2018	MaleSprague-Dawley	OA	MIA	knee joint	EA	ST35ST36	2/10 Hz,1 mA
Shen et al	2021	MaleSprague–Dawley	OA	CFA	ankle joint	MA	ST36	lifting-thrusting,and twisting

Abbreviation: CFA, complete Freund’s adjuvant; CIA, incomplete Freund’s adjuvant; GA, gouty arthritis; MIA, monosodium iodoacetate; MSU, monosodium urate; OA, osteoarthritis; RA, rheumatoid arthritis.

### 3.4 Effect of acupuncture on pain behaviors

**FIGURE 3:**
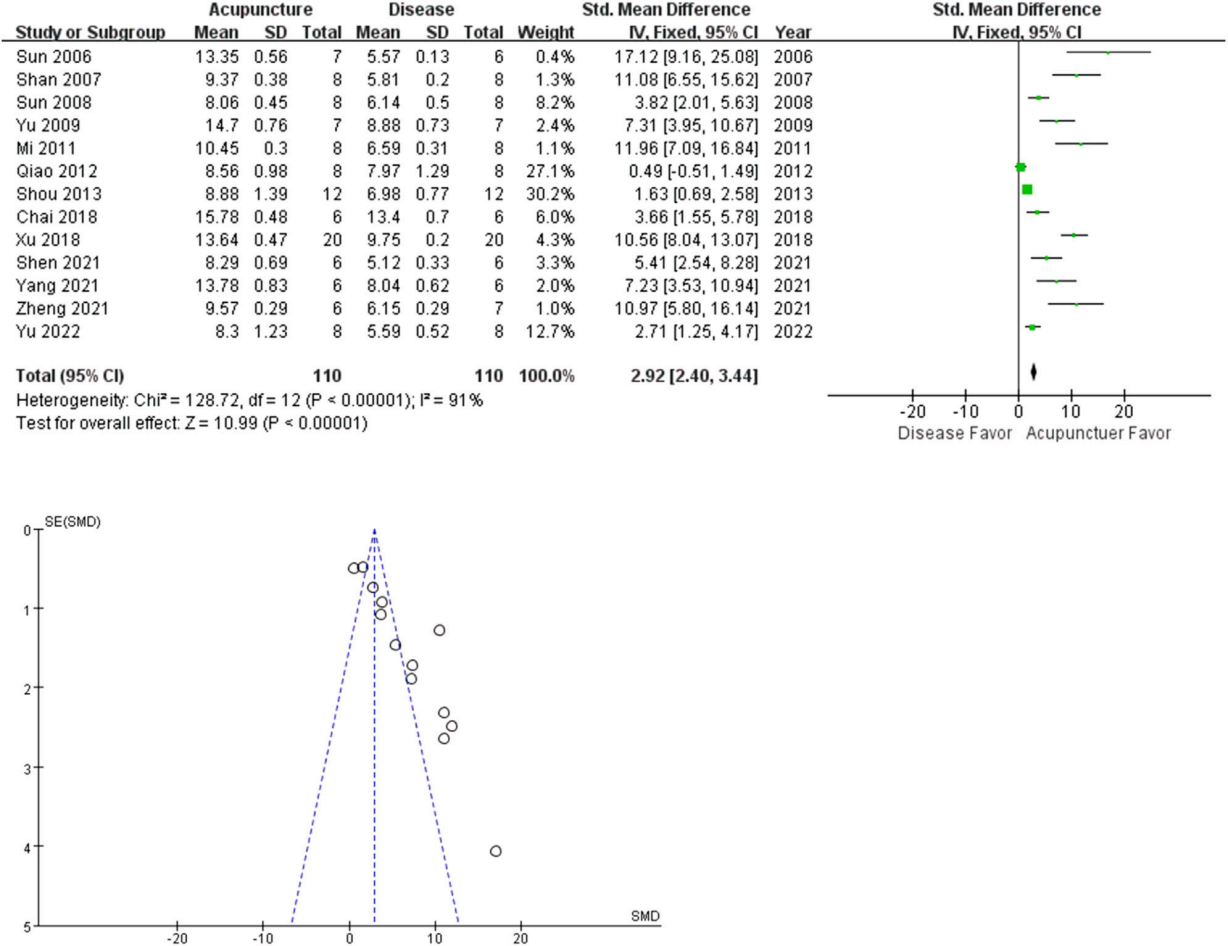
Meta-analysis comparing PWLs between the disease group and the acupuncture-treated group.

**FIGURE 4:**
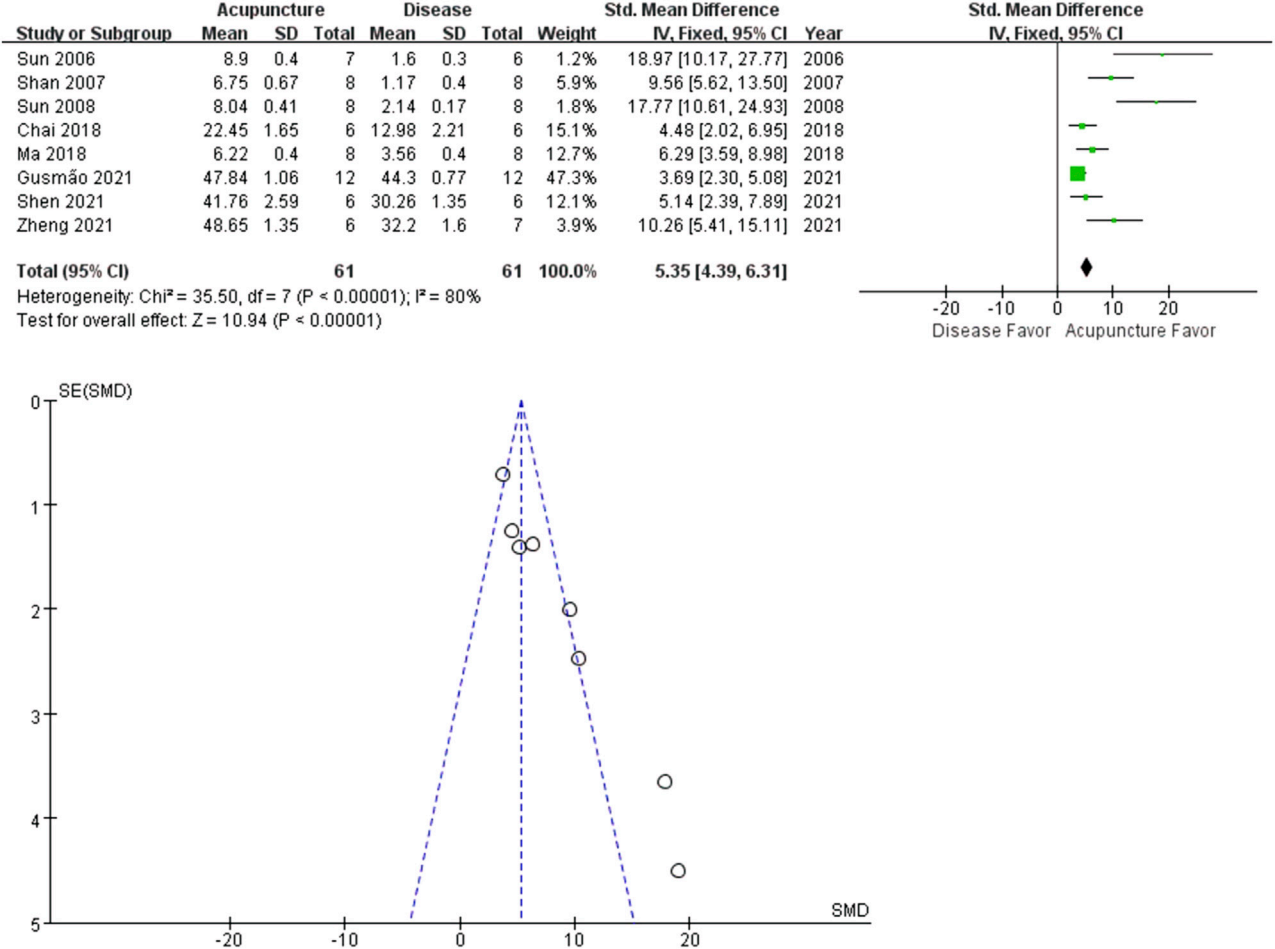
Meta-analysis comparing PWTs between the disease group and the acupuncture-treated group.

The analgesic effect of acupuncture on arthritis was evaluated based on PWL and PWT. Among the 21 studies, 13 studies adopted PWL as an outcome index. The rat arthritis (disease) groups had lower mean PWL values than the acupuncture groups (*n* = 110; SMD = 2.18 [95% CI = 2.40–3.44]; *p* < 0.00001; heterogeneity *X* ^*2*^ = 128.72, *I* ^*2*^ = 91%, Figure 3). A similar trend was observed in the PWT analysis. 9 studies reported the acupuncture treated groups were more tolerant to pressure (*n* = 61; SMD = 5.35 [95% CI = 4.39–6.31]; *p* < 0.00001; heterogeneity *X* ^*2*^ = 35.50, *I* ^*2*^ = 80%, Figure 4).

### 3.5 Effect of acupuncture on swelling

**FIGURE 5:**
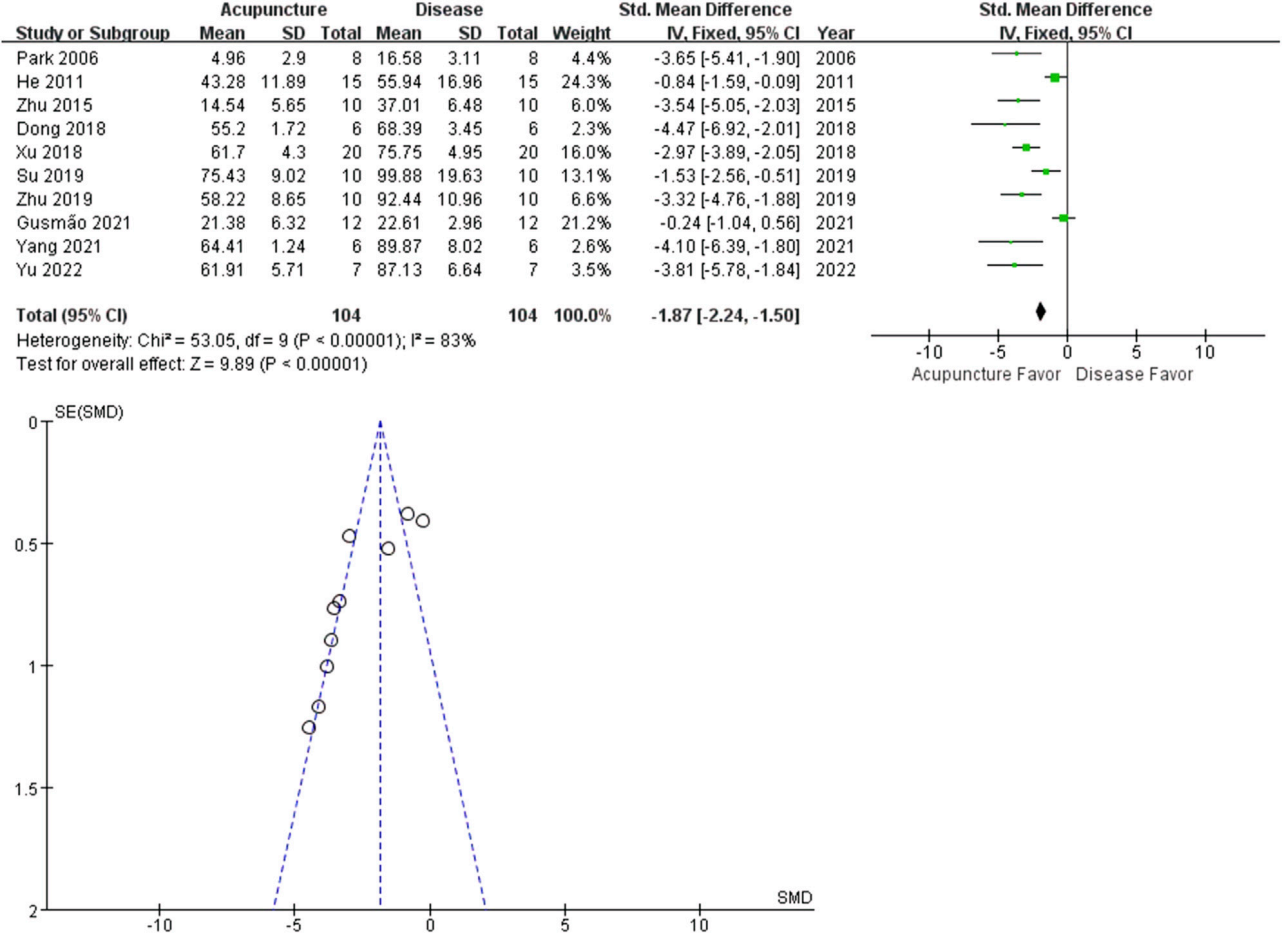
Meta-analysis comparing percentage increase in paw volume between the disease group and the acupuncture-treated group.

Inflammation contributes to arthritis-induced pain. Local swelling is a common immune response in arthritis, associated with increased immune cell infiltration. The percentage increase in paw volume was calculated from the paw volume measurement. Outcomes from the 10 studies revealed that acupuncture reduced hind paw swelling (*n* = 104; SMD = −1.87 [95% CI = −2.24 ∼ −1.50]; *p* < 0.00001; heterogeneity *X* ^*2*^ = 53.05, *I* ^*2*^ = 83%, Figure 5).

### 3.6 Mechanism responsible for the therapeutic action

The symptom scores and mechanisms of each included study were summarized in Table 2. The included studies were classified into either “The analgesic effect of acupuncture” or “The anti-inflammatory effect of acupuncture” based on the focus of the research. 15 studies showed acupuncture reduced pain, as it improved PWL, PWT or weight bearing test in the disease models. Acupuncture was found to reduce swelling (paw volume and ankle diameter) or to improve articular damage scores (pain score and arthritis index score) in 13 studies. One study (Gusmao et al., 2021) examined the effect of acupuncture on osteogenesis (bone radiographic density), where no significance was found between the disease and the acupuncture group.

**TABLE 2:** Symptom score change by acupuncture treatment and the underlying mechanisms.

Author	Year	Symptom score	Biochemical mechanism
The analgesic effect of acupuncture			
Sun et al	2006	PWL △	glial cell (counteract EA effect)
		PWT △ ankle diameter ▽	
Shan et al	2007	PWT △	spinal cord: spinal glial activation ▽
			GFAP, OX-42, IL-1β, IL-6, TNF-α ▽
Sun et al	2008	PWL △	spinal dorsal horn: spinal glial activation ▽
		PWT △ ankle diameter ▽	GFAP, OX-42 ▽
Yu et al	2009	PWL △	N/A
Mi et al	2011	PWL △	spincal cord: NT-3 △
			CD11b, IL-1β, Iba-1, IL-6, TNF-α ▽
Qiao et al	2012	PWL △	hypothalamic paraventricular nucleus: corticotropin release hormone △
Shou et al	2013	PWL △	corpus striatum: CB1 receptor, D1/D2 receptor △
Chai et al	2018	PWL △	local: *ß*-endorphin △ (μ- and κ-opioid receptor-dependent)
		PWT △ ankle diameter ▽	
		ongoing pain score ▽	
Ma et al	2018	PWT △ weight bearing test △	N/A
Shen et al	2021	PWL △	eATP of bilateral isolated lumbar 4–5 dorsal root ganglia ▽
		PWT △	eATP of bilateral sciatic nerves △
Zheng et al	2021	PWL △	TRPV4 activation, eATP △
		PWT △	
The anti-inflammatory effect of acupuncture			
Park et al	2006	paw volume ▽	--
He et al	2011	paw volume ▽	local: VIP △
		body weight △	
Zhu et al	2015		spleen: balanced Treg/Th17 ratio
		paw volume ▽	CD4+FOXP3+ Treg cell △
		arthritis index score ▽	CD4+IL17 + Th17 cell ▽
			VIP receptor △ local: VPAC1 △
Xu et al	2018		local: IL-1β, IL-6, TNF-α▽
		paw volume ▽	serum: IL-1α, IL-7, IL-18 (innate) △
		PWT △	IL-2, IL-12, IFN-γ, IL-4, IL-5, IL-10, IL-13, IL-17 (adaptive) △
Dong et al	2018	arthritis index score ▽	local: TLR4, MYD88, NF-kB. ▽
		paw volume ▽	
Zhu et al	2019	paw volume ▽	local: CD34^+^, HIF-1α, VEGF ▽
		arthritis index score ▽	
Su et al	2019	paw volume ▽	local: apoptosis △
		arthritis index score ▽	p53, Bax, Noxa and PUMA △
		Apoptosis rate △	MDM2 ▽
Gusmão et al	2021	paw volume ▽	local: NF-κB, IL-6, IL-17 ▽
		PWT △ bone radiographic density -	
Yang et al	2021	paw volume ▽	local: IL-1β, IL-4, IL-18 ▽
		PWL △	
Yu et al	2022		local: M1 macrophage ▽ (TGF-β1-dependent)
		paw volume ▽	Treg △
		PWL △	TNF-α, IL-1β ▽
			IL-10, TGF-β1 △

Abbreviation: Bax, Bcl-2-associated X protein; CB1, cannabinoid receptor type 1; D1/D2 receptor, dopamine receptor D1 and D2; GFAP, glial fibrillary acidic protein; HIF, hypoxia-inducible factor; Iba-1, ionized calcium-binding adapter molecule1; IFN-γ, interferon-γ; IL, interleukin; MDM2, Mouse double minute 2 homolog; MYD88, myeloid differentiation factor 88; N/A, not applicable; NF-kB, nuclear factor kappa-B; NT-3, neurotrophin-3; PUMA, p53 upregulated modulator of apoptosis; PWL, pain withdrawal latency; PWT, pain withdrawal threshold; TGF-β1, transforming growth factor; Th, T helper cell; TLR4, Toll-like receptor 4; TNF-α, tumor necrosis factor-alpha; Treg, regulatory T cell; TRPV4, transient receptor potential cation channel subfamily V member 4; VEGF, vascular endothelial growth factor; VIP, vasoactive intestinal peptide; VPAC1, VIP receptor type 1.

In the 11 studies of analgesia, pain behaviors were used as the main symptom score. The mechanisms were related to the nervous system. Among them, 5 studies focused on identifying the neural pathway activated by acupuncture (Sun et al., 2006; Shan et al., 2007; Sun et al., 2008; Mi et al., 2011; Shen et al., 2021); 2 studies identified the brain regions and the responsive proteins for acupuncture-mediated analgesia (Qiao et al., 2012; Shou et al., 2013); one study observed the activity of the opioid system-related molecules (Chai et al., 2018). One study checked transient receptor potential vanilloid-type 4 (TRPV4) activity during acupuncture treatment (Zheng et al., 2021).

All the 10 studies of anti-inflammation included paw volume measurement. Various mechanisms have been proposed in this section. Most of the studies focused on the immune regulation: changes in cytokine levels were measured in 4 studies (Xu et al., 2018; Gusmao et al., 2021; Yang et al., 2021; Yu et al., 2022); NF-kB signaling was investigated in 2 studies (Dong et al., 2018; Gusmao et al., 2021); changes in immune cell proportions were assessed in 2 studies (Zhu et al., 2015; Yu et al., 2022). Expression levels of vasoactive intestinal peptide (VIP) and its binding receptors were measured in 2 studies (He et al., 2011; Zhu et al., 2015); VIP is a neuropeptide with pleiotropic functions, like vasodilation and immune regulation (Delgado and Ganea, 2013). Apoptotic rates and the associated protein levels were measured in one study (Su et al., 2019). Synovial angiogenesis was examined in one study (Zhu et al., 2019).

## 4 Discussion

Recently, acupuncture analgesia was systemically reviewed in various clinical conditions (Nielsen and Wieland, 2019). Cochrane reviews suggest acupuncture is probably effective in peripheral joint osteoarthritis. We performed a meta-analysis on PWL (13 studies), PWT (8 studies) and percentage increase in paw volume (10 studies), to determine the analgesic and the anti-inflammatory effects of acupuncture on hind limb arthritis. All the included studies reported the positive effects of acupuncture on arthritis-induced pain and swelling. The total SMDs of both PWL and PWT were larger in the acupuncture group, meaning increased tolerance to stimuli. PWL and PWT are behavioral methods to measure stimulus-evoked pain threshold (hyperalgesia or allodynia). On the other hand, acupuncture decreased percentage increase in paw volume of arthritis rats. Percentage increase in paw volume is used to estimate the effectiveness of anti-inflammatory treatment to reduce edema. Our results suggest acupuncture may be more effective in reducing pain than swelling. The small had larger effect sizes than the larger studies, the funnel plot asymmetry seen in this review could be a generic means of displaying small-study effects.

**FIGURE 6:**
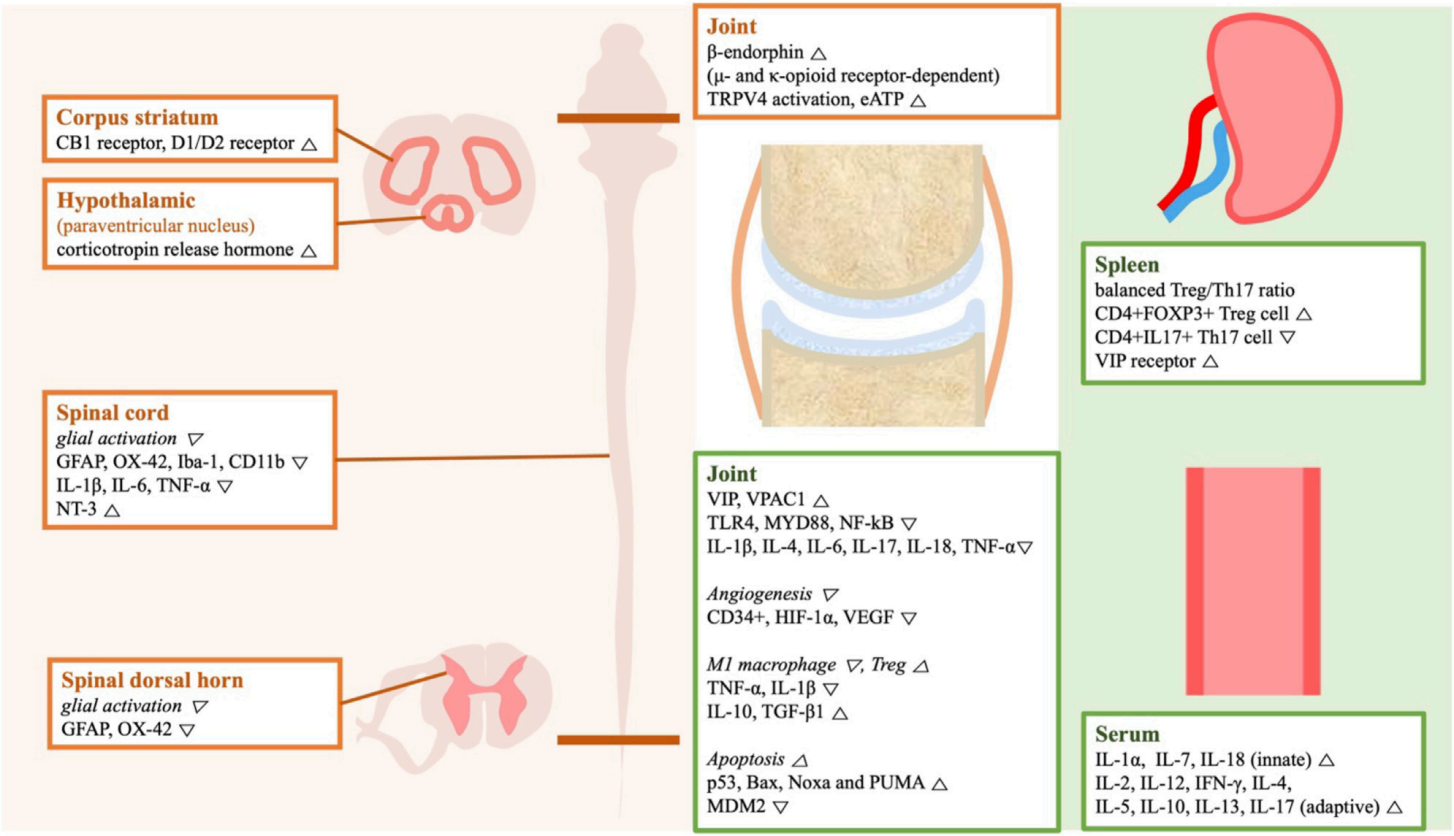
A schematic illustration of the genes and the signaling molecules regulated by acupuncture treatment in the included animal studies. The gene expression listed in orange boxes is related to acupuncture (left), while those in green boxes are related to acupuncture-mediated anti-inflammation. Abbreviation: Bax, Bcl-2-associated X protein; CB1, cannabinoid receptor type 1; D1/D2 receptor, dopamine receptor D1 and D2; GFAP, glial fibrillary acidic protein; HIF, hypoxia-inducible factor; Iba-1, ionized calcium-binding adapter molecule1; IFN-γ, interferon-γ; IL, interleukin; MDM2, Mouse double minute 2 homolog; MYD88, myeloid differentiation factor 88; N/A, not applicable; NF-kB, nuclear factor kappa-B; NT-3, neurotrophin-3; PUMA, p53 upregulated modulator of apoptosis; PWL, pain withdrawal latency; PWT, pain withdrawal threshold; TGF-β1, transforming growth factor; Th, T helper cell; TLR4, Toll-like receptor 4; TNF-α, tumor necrosis factor-alpha; Treg, regulatory T cell; TRPV4, transient receptor potential cation channel subfamily V member 4; VEGF, vascular endothelial growth factor; VIP, vasoactive intestinal peptide; VPAC1, VIP receptor type 1.

A wide spectrum of mechanisms was introduced to explain the therapeutic effects of acupuncture (Figure 6). The studies of analgesia focused on the nervous system. 4 studies reported downregulated glial cell activity in spinal cords of CFA-induced RA rats upon GB30 and GB34 stimulation (Sun et al., 2006; Shan et al., 2007; Sun et al., 2008; Mi et al., 2011). The ST36 and BL60 stimulation upregulated the peripheral opioid system and the central dopamine system (Shou et al., 2013; Chai et al., 2018). 2 studies focused on accumulation of extracellular adenosine (Shen et al., 2021; Zheng et al., 2021); adenosine regulates pain transmission in both the central and peripheral nervous system. One of the studies suggests TRPV4 to be crucial for extracellular adenosine accumulation upon MA treatment. TRPV4 is a mechanosensitive cation channel expressed in different cell types including innate immune cells and parenchymal cells (Michalick and Kuebler, 2020).

There has been progress in surface-level mechanism research on the analgesic effects of acupuncture. However, more multidisciplinary research is needed. Considering that acupuncture was mainly developed in East Asia, the database on its efficacy across different human races is still insufficient. Furthermore, acupuncture’s effects may vary between individuals, and how genetic background may contribute to such variation remains in question. In the case of acupuncture analgesia, various receptors are suggested to be involved in pain relief; different genetic variations in neural receptors like CB1 and D1/D2 receptors may be related to diverse pain patterns among patients. Additionally, there have been many recent efforts to link the nervous and immune systems, and TRPV4 is an example of this (Michalick and Kuebler, 2020). Certain mutations in TRPV4 cause several neuromuscular and skeletal disorders (McCray et al., 1993). Through research that associates the analgesic effect of acupuncture with genetic background, it may be helpful to identify patients susceptible to acupuncture treatment and expand the range of patients who could benefit from acupuncture treatment.

Genetic backgrounds determine the severity of certain types of arthritis. Some HLA genotypes are particularly associated with more aggressive forms of rheumatoid arthritis (Smolen et al., 2016). More than a hundred loci have been characterized to be relevant to arthritis risk, while many of them are related to immune regulation (Smolen et al., 2016; Aubourg et al., 2022). Genetic investigation of arthritis is on progress to provide better translational intervention. Considering acupuncture treatment generally works best in the early stages of arthritis, the utilization of such genetic knowledge may conduce to optimizing treatment regimens of acupuncture for arthritis.

Acupuncture treatment tends to lower the pro-inflammatory cytokine (e.g., TNF-α, IL-1β, IL-4, IL-6, IL-17, IL-18) levels at the inflammatory sites (Xu et al., 2018; Gusmao et al., 2021; Yu et al., 2022). In consistent with these findings, NF-κB signaling was inhibited by acupuncture (Dong et al., 2018; Gusmao et al., 2021). Acupuncture also seems to promote the pro-resolving inflammation by reducing M1 macrophage polarization and increasing regulatory T cell population (Zhu et al., 2015; Yu et al., 2022). Other factors like increased apoptosis and inhibited angiogenesis may also help to control inflammation (Su et al., 2019; Zhu et al., 2019). VIP and its receptors were studied by two teams, while how VIP reduces inflammation remains unsolved (He et al., 2011; Zhu et al., 2015).

All the studies used reagent-induced arthritis models. Two reagents were used in RA models: CFA is a mixture of emulsifying agent, mineral oils, and heat-killed mycobacteria; CIA contains cartilage-derived collagen. The genetic background of rats is important in both models, as major histocompatibility complex (MHC) contributes to the susceptibility to arthritis (Choudhary et al., 2018). CFA is powerful enhancer of both cell-mediated and humoral immune responses, and repeated immunization can lead to permanent joint malformation; in rats, TNF-α, IL-1β and IL-17 are present throughout the disease progression, while macrophage-stimulating cytokines, IL-4, IL-6, and TGF-β are detected in severe cases. T helper cell activation is more prominent in rat CIA models, with TNF-α and IL-1β to be the key cytokines. MIA-induced OA in rats is popular particular in pain research, due to its rapidity and reproducibility (Kuyinu et al., 2016). The circulating monocytes and macrophages seem to be the initiators of low-grade inflammation in MSU-induced gouty arthritis (Cabau et al., 2020).

Despite the heterogeneity of arthritis models, acupoints located below the knee joint were frequently used (GB34, GB39, SP6, ST35, ST36, ST44), where most studies concluded stimulating these acupoints to be effective in symptom improvement. Interestingly, other factors are found to enhance treatment effects of acupuncture on arthritis symptoms. EA than MA, ipsilateral than contralateral, and early treatment increased the pain threshold of arthritis rats more efficiently (Sun et al., 2006; Shan et al., 2007; Sun et al., 2008; Yu et al., 2009; Ma et al., 2018). When using EA, low-frequency (2 Hz) or alternate 2/100 Hz gave greater improvement in arthritis-induced pain (Park et al., 2006; Chai et al., 2018).

There are limitations to the present study. First, the disease models and experiment designs of the included studies vary, e.g., injection sites, experiment timelines, cut off points for behavioral tests etc., as this review aims to encompass overall effects of acupuncture on arthritis in rats. Second, the number of included studies is inadequate for making a complete conclusion about the efficacy of acupuncture on arthritis. However, this study is meaningful in that it is the first systematic review to assess the pain-relieving and anti-inflammatory effects of acupuncture in arthritis using animal models, by analyzing PWL, PWT and percentage increase in paw volume. We also offered a snapshot of the current understanding on acupuncture-mediated analgesia and immunomodulation in animal models.

In summary, acupuncture might ameliorate arthritis-induced pain and swelling by regulating the nervous and immune system. We hope that the current review will be a good reference for ongoing research of this field.

## Data Availability

The original contributions presented in the study are included in the article, further inquiries can be directed to the corresponding author.
